# Can the frontal tibiofemoral alignment be assessed on anteroposterior knee radiographs?

**DOI:** 10.1007/s10195-016-0404-0

**Published:** 2016-04-16

**Authors:** M. Sgroi, M. Faschingbauer, H. Reichel, T. Kappe

**Affiliations:** Department for Orthopaedic Surgery, University of Ulm, Oberer Eselsberg 45, 89081 Ulm, Germany

**Keywords:** Knee arthroplasty, Alignment, Full strut, AP knee recording

## Abstract

**Background:**

The aim of total knee arthroplasty is, amongst others, the reconstruction of a physiological axis of the leg with a tibiofemoral angle in the frontal plane of an average of 6°. The aim of this study is to clarify how much of the bone length on the femur and tibia has to be reproduced on anteroposterior (AP) knee radiographs in order to determine the leg’s alignment after a total knee arthroplasty.

**Materials and methods:**

We analyzed the postoperative hip-to-ankle (HTA) radiographs of 100 patients who had undergone a total knee arthroplasty at our institution.

**Results:**

There were strong correlations between the measured values on HTA and 20 cm bone length [lateral distal femur angle (LDFA) *r* = 0.887, medial proximal tibial angle (MPTA) *r* = 0.874, tibiofemoral angle (TFA) *r* = 0.888], but not between the measurements on HTA and 10 cm (LDFA *r* = 0.267, MPTA *r* = 0.102, TFA *r* = 0.161). There were significant differences between all measurements both on HTA and 20 cm and on HTA and 10 cm, with the exception of the LDFA between HTA and 10 cm (*p* = 0.085) and of the MPTA between HTA and 20 cm (*p* = 0.227). The intra- and inter-observer correlations were both high.

**Conclusion:**

If preoperatively crude axis deviations are excluded, the tibiofemoral angle on AP knee radiographs can be determined with an accuracy of ±2.6° if at least 20 cm length of bone is reproduced (measured from the femoral and tibial joint line). Due to the high 95 % confidence intervals and bearing in mind that deviations greater than 3° may lead to inferior clinical results, however, it appears inappropriate to determine lower limb alignment with anteroposterior radiographs.

**Level of evidence:**

Level 2.

## Introduction

The traditional aim of total knee arthroplasty (TKA) is to restore a neutral axis of the leg with a tibiofemoral angle (TFA) of 5°–7° [14]. Malalignment after TKA, especially in reference to the tibial component, has been shown to lead to negative biomechanical as well as clinical consequences, e.g., early loosening [11]. The TFA is traditionally measured on hip-to-ankle (HTA) radiographs. This is used both for preoperative surgical planning and to verify the correct reconstruction of the postoperative axis. The HTA has been shown to be prone to errors in rotation and location of the central beam as well as insufficient weight application [8]. Even though recent studies confirm that it is not possible to derive the TFA from short knee radiographs [4], it is still unclear how much of the femur and the tibia has to be depicted in order to measure the TFA accurately. The aim of this study, therefore, was to determine what bone length of the femur and tibia has to be exposed in order to determine the TFA correctly.

## Materials and methods

Between August 2010 and December 2011, 428 consecutive patients with degenerative osteoarthritis of the knee underwent primary TKA at our department. HTA radiographs were done preoperatively in all patients. Patients meeting the following criteria were excluded from the study: (1) a history of fracture of the lower limb; (2) a history of lower limb axis correction surgery; (3) crude axis deviation (excessive femur varum or curvature of the tibia); and (4) for technical reasons (e.g., malrotated X-ray). After exclusion of these criteria, 100 HTA radiographs were selected at random and included in the present study (57 women and 43 men). All the HTA radiographs investigated were performed between days 3 and 7 after surgery. The average age of the patients was 68.6 years (range 49–86 years).

**Fig. 1:**
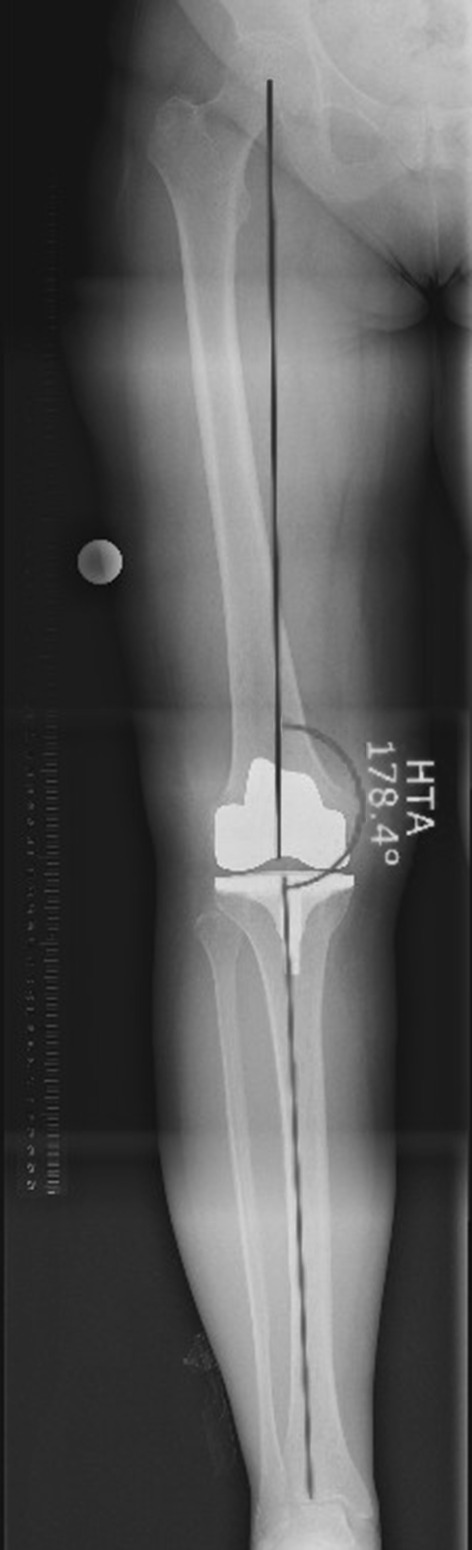
Measurement of HTA angle (*HTA* angle between the mechanical axis of the femur and mechanical axis of the tibia)

All the recordings analyzed were HTA radiographs obtained on graduated-grid 30 × 90 cm cassettes. The recordings were performed in standing position and in full extension at a distance of 3 meters (Fig. 1). Reference bodies of 2.5 cm diameter were routinely attached to the patient’s skin at the level of the knee. Rotation was controlled by determining the amount of the superposition of fibular head and lateral tibial plateau. Radiographs were deemed acceptable if 1/3 of the fibular head was superimposed. Malrotated radiographs were excluded from the study.

**Fig. 2:**
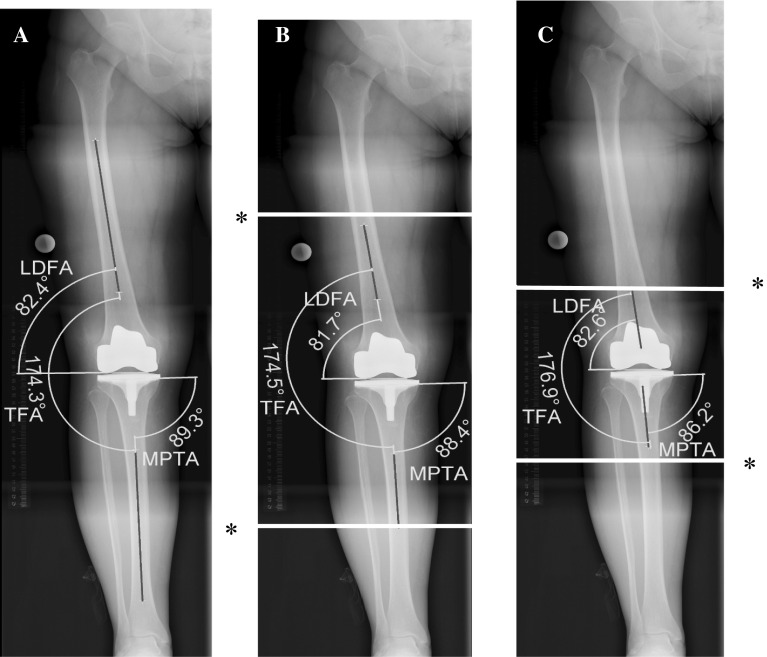
Measurement of TFA, MPTA and LDFA on **a** HTA radiograph, **b** 20 cm bone length radiograph, **c** 10 cm bone length radiograph. *TFA* tibial femoral angle, *LDFA* lateral distal femur angle, *MPTA* medial proximal tibial angle, *HTA* hip-to-ankle radiograph

First, femoral component orientation (lateral distal femur angle, LDFA) was determined by measuring the angle between a line connecting the most distal points on the surfaces of the femoral condyles and a line connecting the centre of the femoral canal at two points 30 cm apart, according to Paley [12]. Tibial component orientation (medial proximal tibial angle, MPTA) was then measured as the angle between a line connecting the most proximal medial and proximal lateral points of the tibial component to a line connecting the centre of the tibial medullary canal at two points 30 cm apart. The TFA was determined as the angle between the two intramedullary axes described above. HTA radiographs were then imaginarily cut at intervals of 20 and 10 cm length of bone, measured from the joint line, on both the femoral and the tibial sides. The same measurements as above were then carried out after every imaginary shortening and the above parameters measured again (Fig. 2).

In order to allow determination of the intra-observer and inter-observer reliability, 20 HTA radiographs were examined twice by two orthopaedic surgeons. The survey was carried out using the AGFA planning system (Agfa HealthCare GmbH, Bonn, Germany).

The collected data were analyzed using Pearson’s correlation coefficient (PCC) using simple linear regression, the *t* test and 95 % confidence intervals. The differences were considered significant if the *p* value was less than 0.05. The statistical analysis was performed using SSPS.

## Results

The results of the measurements are presented in Table 1. There was a strong correlation between the measured values on HTA and 20 cm bone length (LDFA *r* = 0.887, *p* < 0.001; MPTA *r* = 0.874, *p* < 0.001; TFA *r* = 0.888, *p* < 0.001), but not between the measurements on HTA and 10 cm (LDFA *r* = 0.267 *p* = 0.007, MPTA *r* = 0.102 *p* = 0.314, TFA *r* = 0.161 *p* = 0.109). There were significant differences between all measurements on HTA and 20 cm and on HTA and 10 cm, with the exception of the LDFA between HTA and 10 cm (*p* = 0.085) and the MPTA between HTA and 20 cm (*p* = 0.223). The 95 % confidence interval for the LDFA was 2.0° (HTA and 20 cm) and 5.5° (HTA and 10 cm), for the MPTA 1.7° (HTA and 20 cm) and 5.1° (HTA and 10 cm) and for the TFA 2.6° (HTA and 20 cm) and 7.8° (HTA and 10 cm). The inter-observer correlation was high (Table 2).

**Table 1:** Measurement of TFA, MPTA and LDFA on HTA and on both short anteroposterior knee radiographs

	HTA	20 cm bone length	10 cm bone length
TFA	173.23 ± 2.86	172.91 ± 2.60	176.09 ± 3.29
LDFA	83.88 ± 2.19	83.61 ± 1.84	84.37 ± 2.39
MPTA	90.75 ± 1.74	90.85 ± 1.51	88.38 ± 2.19

*TFA* tibial femoral angle, *LDFA* lateral distal femur angle, *MPTA* medial proximal tibial angle, *HTA* hip-to-ankle radiograph

**Table 2:** Inter-observer reliability for TFA, MPTA and LDFA on HTA and on both short anteroposterior knee radiographs

	HTA	20 cm bone length	10 cm bone length
TFA	0.926*	0.882*	0.729**
LDFA	0.918*	0.842*	0.844*
MPTA	0.892*	0.841*	0.892*

*TFA* tibial femoral angle, *LDFA* lateral distal femur angle, *MPTA* medial proximal tibial angle, *HTA* hip-to-ankle radiograph

* *p* > 0.001, ** *p* > 0.003

## Discussion

The aim of the current study was to determine the length of femur and tibia which has to be reproduced on anteroposterior radiographs in order to accurately measure lower limb alignment. The results suggest that exposing 20 cm of the femur and tibia will introduce a rather negligible measurement error of −2.6 to 2.6°.

Determining alignment before and after TKA is essential for surgical planning, execution of the operation and postoperative evaluation of the treatment result [2]. Different studies have shown that a postoperative malalignment contributes to reducing the longevity of TKA [3].

Both short anteroposterior knee radiographs and HTA radiographs are used to check lower limb alignment after TKA. Short knee radiographs do not reveal extra-articular deformity or deviations in femoral neck length, resulting in 6° ± 1° angle deviation. Therefore, only the anatomical, not the mechanical axis can be derived from short knee radiographs. In a study by McGrory et al. [10] it was shown that the mechanical axis plays a rather subordinate role in comparison to the TFA in axis reconstruction.

The concept of neutral alignment after TKA has recently been challenged, however. In a study by Matziolis et al. [9] it was postulated that a varus malalignment after TKA has no effect on clinical outcome. In another study by Bellemans [1] it was proposed to re-establish the patient’s own native baseline type of alignment. In comparison to the predominance of data supporting a neutral mechanical axis and approximately 5°–7° valgus anatomic alignment, there is little support for choosing any other aim [7].

There is a paucity of data on whether the TFA can be determined with sufficient accuracy on postoperative anteroposterior knee radiographs. In 1988, Petersen et al. compared the TFA measured on anteroposterior knee radiographs with measurements on HTA radiographs in 50 patients after total knee arthroplasty and found a discrepancy of 1.4° with a standard deviation (SD) of 2.2° [13]. In a similar study on 83 patients, Skyttä et al. [15] measured a difference of 1.4° with an SD of 1.4° for the TFA. The two studies have shown that both TFA measurement techniques correlate strongly with each other.

Hirschmann et al. [5] measured the intra- and inter-observer reliability of measurements of the position of the components after total knee replacement using plane radiographs and axial two-dimensional and three-dimensional reconstructed computed tomography (CT) images. They found that three-dimensional reconstructed images are sufficiently reliable to measure the position and orientation of the components. The derivation of postoperative alignment after TKA using CT is associated with a higher radiation exposure for the patient, and for this reason this method cannot be considered a standard procedure.

The present study has several limitations. First, outliers were preliminarily prevented by excluding patients with gross alignment deviation. This appeared intuitive to us as we felt this would increase the clinical applicability of the data. Even if HTA radiographs were not acquired on a routine basis, they would at least be obtained in patients posing challenges for intraoperative limb alignment correction. Second, we artificially cut HTA films to short radiographs in order to prevent having our results affected by additional deviations in projection and rotation. In clinical practice, the differences between shorter and HTA radiographs may therefore be even higher than the results of the present study suggests. We were interested in the magnitude of the effect of limiting exposure on measurement accuracy, and artificially cutting HTA films appeared to perfectly exclude further influencing factors while limiting unnecessary X-ray exposure to patients.

In summary, this study shows that it is theoretically possible to derive lower limb alignment from shorter knee radiographs if at least 20 cm of the tibia and femur are depicted and if gross extra-articular deformity has previously been excluded. However, this study also suggests that a measurement error of −2.6° to 2.6° already arises by the exposure of 20 cm of the femur and the tibia. Due to these high 95 % confidence intervals and bearing in mind that deviations greater than 3° may lead to inferior clinical results [6], however, it appears inappropriate to determine lower limb alignment with shorter knee radiographs. Therefore, we do not recommend determining lower limb alignment with short anteroposterior radiographs; HTA radiographs should be considered the gold standard for routine practice.
